# CSF/plasma HIV-1 RNA discordance even at low levels is associated with up-regulation of host inflammatory mediators in CSF^☆^

**DOI:** 10.1016/j.cyto.2016.04.004

**Published:** 2016-07

**Authors:** Sam Nightingale, Benedict D. Michael, Martin Fisher, Alan Winston, Mark Nelson, Steven Taylor, Andrew Ustianowski, Jonathan Ainsworth, Richard Gilson, Lewis Haddow, Edmund Ong, Clifford Leen, Jane Minton, Frank Post, Apostolos Beloukas, Ray Borrow, Munir Pirmohamed, Anna Maria Geretti, Saye Khoo, Tom Solomon

**Affiliations:** a Institute of Infection and Global Health, University of Liverpool, UK; b Department of Molecular and Clinical Pharmacology, University of Liverpool, Liverpool, UK; c Royal Liverpool and Broadgreen University Hospitals NHS Trust, UK; d Walton Centre for Neurology and Neurosurgery, Liverpool, UK; e Brighton and Sussex University Hospitals NHS Trust, UK; f St Marys’ Hospital, Imperial College Heathcare NHS Trust, London, UK; g St Stephen’s AIDS Research Trust and Chelsea and Westminster Hospital NHS Foundation Trust, UK; h Birmingham Heartlands Hospital, Heart of England NHS Foundation Trust, UK; i North Manchester General Hospital, Pennine Acute Hospitals NHS Trust, UK; j North Middlesex University Hospital NHS Trust, UK; k Research Department of Infection and Population Health, University College London, UK; l Victoria Royal Infirmary, Newcastle upon Tyne Hospitals NHS Trust, UK; m Royal Infirmary of Edinburgh, NHS Lothian, UK; n Leeds General Infirmary, Leeds Teaching Hosptials NHS Trust, UK; o Kings College Hospital NHS Foundation Trust, London, UK; p Vaccine Evaluation Unit at the Health Protection Agency (HPA) North West, Manchester, UK

**Keywords:** HIV, Cerebrospinal fluid, CSF escape, Sanctuary site, Inflammation

## Abstract

**Introduction:**

HIV-1 RNA can be found at higher levels in cerebrospinal fluid (CSF) than in plasma, termed CSF/plasma discordance. The clinical significance of CSF/plasma discordance is not known and the degree of discordance considered important varies. We aimed to determine whether a panel of CSF cytokines, chemokines and associated mediators were raised in patients with CSF/plasma discordance at different levels.

**Methods:**

A nested case-control study of 40 CSF samples from the PARTITION study. We used a cytometric bead array to measure CSF mediator concentrations in 19 discordant and 21 non-discordant samples matched for plasma HIV-1 RNA. Discordant samples were subdivided into ‘high discordance’ (>1log_10_) and ‘low discordance’ (0.5–1log_10_, or ultrasensitive discordance). CSF mediators significant in univariate analysis went forward to two-way unsupervised hierarchical clustering based on the patterns of relative mediator concentrations.

**Results:**

In univariate analysis 19 of 21 CSF mediators were significantly higher in discordant than non-discordant samples. There were no significant differences between samples with high versus low discordance. The samples grouped into two clusters which corresponded to CSF/plasma discordance (p < 0.0001). In cluster one all mediators had relatively high abundance; this included 18 discordant samples and three non-discordant samples. In cluster two all mediators had relatively low abundance; this included 18 non-discordant samples and one non-discordant sample with ultrasensitive discordance only.

**Conclusions:**

CSF/plasma discordance is associated with potentially damaging neuroinflammatory process. Patients with discordance at lower levels (ie. 0.5–1log_10_) should also be investigated as mediator profiles were similar to those with discordance >1log_10_. Sensitive testing may have a role to determine whether ultrasensitive discordance is present in those with low level CSF escape.

## Highlights

• Discordant HIV in CSF is associated with raised inflammatory mediators in CSF.
• CSF mediators are raised with discordance both at high and low levels.
• Discordance on ultrasensitive testing can also be also associated with raised mediators.

## 1 Introduction

For some patients with human immunodeficiency virus (HIV) viral ribonucleic acid (RNA) is at higher levels in cerebrospinal fluid (CSF) than plasma [1], [2]. This phenomenon, termed CSF/plasma discordance, can occur in approximately 10% of individuals with HIV undergoing a lumbar puncture in clinical practice [1], [2], [3], [4]. The causes and clinical significance of discordance remain to be determined. Whilst CSF/plasma discordance has been associated with fulminant central nervous system (CNS) diseases in case series, these appear to be uncommon in clinical practice [5], [6]. Moreover, the association of CSF/plasma discordance with cognitive impairment in antiretroviral therapy (ART) treated cohorts is not clear [7], [8], [9]. The pathophysiological processes underlying discordance are also not clear, in particular it is not known if CSF/plasma discordance is associated with an up-regulation in the host inflammatory response and at what level of discordance this occurs. Previous studies have identified up-regulation of several cytokines and associated mediators in patients with cognitive decline although this has not been investigated in discordance [10], [11].

There is currently no consensus on the degree of discordance between CSF and plasma HIV-1 RNA that should be considered clinically significant. Some studies have defined discordance as CSF HIV-1 RNA 1log_10_ greater than the corresponding plasma sample [5], whilst others have used a lower cut off of 0.5log_10_ [1]. In those with unquantifiable HIV-1 RNA in plasma (typically <40 or <50 copies/ml) some studies have described the detection of CSF virus at any level above this detection limit as being significant (termed “CSF viral escape”) [2], while others have excluded those with CSF HIV-1 RNA below 200 copies/ml [1], [5]. This has led to difficulties comparing studies as well as confusion in clinical practice as to what cut-off to use.

Sensitive testing can now detect HIV-1 RNA below the traditional threshold of 40 or 50 copies/ml. A proportion of patients have CSF/plasma discordance detected on these assays that would not otherwise be classified as discordant if tested with standard assays [3], [12]. The source and significance of CSF/plasma discordance at this low level remains controversial [13], [14]. Such sensitive testing is currently only available as a research tool, however assays with a lower limit of quantification of 20 copies/ml are now being used routinely in many clinical centres and consequently CSF/plasma discordance below 50 copies/ml is an increasingly recognised phenomenon in clinical practice.

This nested case-control study aimed to assess whether CSF/plasma discordance was associated with cytokines, chemokines and accompanying mediators in the CSF, when compared with non-discordant samples. Furthermore we aimed to examine the relationship between CSF mediator profile and degree of CSF/plasma discordance to determine whether discordance at lower levels (ie levels that have been excluded in some published studies) are associated with potentially damaging CNS inflammation.

## 2 Methods

### 2.1 Study population

Patients were recruited prospectively through the PARTITION study, which is a multicentre UK study described elsewhere [15]. HIV-1 positive ART treated adults were recruited in two groups: 113 patients undergoing lumbar puncture for a clinical indication of which 92 (81%) had no evidence of CNS infection (clinical group), and 40 patients undergoing research lumbar puncture to investigate intermittent or persistent plasma HIV-1 RNA detection (low level viraemia (LLV) group). In this group median HIV-1 RNA levels during viraemic episodes was 92 copies/ml (IQR 59, 179). A randomly selected subgroup with unquantifiable plasma HIV-1 RNA on the standard testing (ie. <40 copies/ml) underwent sensitive HIV-1 RNA detection with a modified Abbott assay with a lower limit of detection of 1–10 copies/ml dependent on volume tested [15].

Patients in the LLV group underwent a brief cognitive screen using the International HIV Dementia scale [16], and were assessed for symptoms of cognitive impairment using the three standard questions described by Simioni et al. [17]. A score of less than ten on the HIV dementia scale was considered abnormal and a patients were considered to have cognitive symptoms when answering “yes, definitely” on at least one of the three questions.

### 2.2 Laboratory assessment

CSF cell count, protein and glucose measurements, microbiological investigations, and CD4 cell counts were performed by routine methods at the centres of care. As lumbar puncture was performed for research purposes in the LLV group, local CSF testing was only performed in this group if considered clinically indicated by the treating physician. HIV-1 RNA was measured centrally in simultaneous plasma and CSF samples by the Abbott RealTime HIV-1 assay (Maidenhead, UK) with a lower limit of quantification of 40 copies/ml, as previously described [18].

### 2.3 Selection criteria

Discordant samples were selected from the clinical and LLV groups. In the clinical group patients with CNS infection were excluded. Discordance was defined as CSF HIV-1 RNA >0.5log_10_ times that measured in a simultaneous plasma sample. In those with unquantifiable plasma HIV-1 RNA, discordance was defined as a CSF HIV-1 RNA >0.5log_10_ times the lower limit of assay quantification (ie. 40 copies/ml). Samples were categorised as “high discordance” if CSF HIV-1 RNA was >1log_10_ times that of plasma and “low discordance” if the ratio was 0.5–1log_10_. Samples with discordance only detected on sensitive assay were termed ‘ultrasensitive discordance’ and analysed within the low discordant group.

For controls, we screened all participants in the PARTITION study who did not have discordance, and selected those with a plasma HIV-1 RNA which most closely matched that of samples with discordance. Those with the lowest CSF HIV-1 RNA were selected.

CSF viral escape was defined as plasma HIV-1 RNA less than the lower limit of quantification for the standard assay (ie 40 copies/ml), and CSF ⩾40 copies/ml.

### 2.4 Measurement of mediators

CSF was collected at recruitment and centrifuged to remove cells. Samples were stored at −70 °C and freeze thaw cycles minimised. Thirty-seven mediators (cytokines, chemokines and associated mediators) were assessed using a cytometric bead array assay system in accordance with the manufacturers’ instructions (Procarta ® Immunoassay kit, Panomic Solutions, Affymetrix ® Milano, Italy. The mediators assessed were chosen from reviewing previous literature: granulocyte-colony stimulating factor [G-CSF], granulocyte-macrophage-colony stimulating factor [GM-CSF], myeloperoxidase [MPO], CCL2 [monocyte chemotactant protein 1], CCL3 [C-C motif ligand 3, monocyte inflammatory protein 1a], CCL4, CCL5 [regulated on activation normal T cell expressed and secreted- RANTES], CXCL10 [inducible protein 10], vascular cell adhesion molecule [VCAM], intracellular adhesion molecule [ICAM], vascular endothelial growth factor [VEGF]α, and matrix metalloproteinases [MMP] 1, 2, 3, 7, 8, 9, 12 and 13, interferon [IFN] α, β, ο, *γ*, tumour necrosis factor [TNF]α and its soluble receptors [TNFR1 and TNFR2] and TNFα-related apoptosis inducing ligand [TRAIL], interleukin [IL] 1α, 1β, 2, 4, 6, 8, 10 and 17a, IL17f, and IL1-receptor antagonist [IL1RA]. Analytes were assessed for all patient groups on one plate for MMPs and one plate for all other mediators. Fluorescence intensity was determined using a Bio-Rad platform (BioPlex Manager 4.1, Bio-Rad Laboratories ©, Hertfordshire, UK). Standards and samples were analysed in duplicate and the mean value used in analysis. Standard curves were adjusted at the points of fluorescence intensity saturation, to generate a sigmoid standard curve with 6–8 fluorescence intensity points. To avoid undetectable levels of mediators or missing data biasing the analysis, only mediators that were detected in at least 80% of the cohort were included in the analyses [19], [20].

### 2.5 Univariate statistical analysis

Clinical and demographic variables were compared by the Mann-Whitney *U* test if continuous and by Chi squared or Fisher’s exact test if categorical. Median concentrations between groups were compared with the Mann-Whitney *U* test. Within cases, individual CSF mediator concentrations were correlated with CSF HIV-1 RNA using Spearman’s r.

### 2.6 Two-way unsupervised multivariate analysis

CSF mediators significantly (p < 0.05) associated with CSF/plasma discordance in univariate analysis were included in a 2-way unsupervised hierarchical cluster analysis based on the patterns of relative mediator concentrations. This offered an opportunity to examine the interactions of mediators and allow samples to be classified by their overall host mediator response. Samples and then mediators were hierarchically clustered using the program Cluster (version 3.0), using the uncentered correlation score as the similarity metric and the average branch linkage method. Results were viewed as heat-maps using Tree View software (http://rana.lbl.gov/EisenSoftware.htm) as described previously [19], [21].

## 3 Results

### 3.1 Subject characteristics

**Fig. 1:**
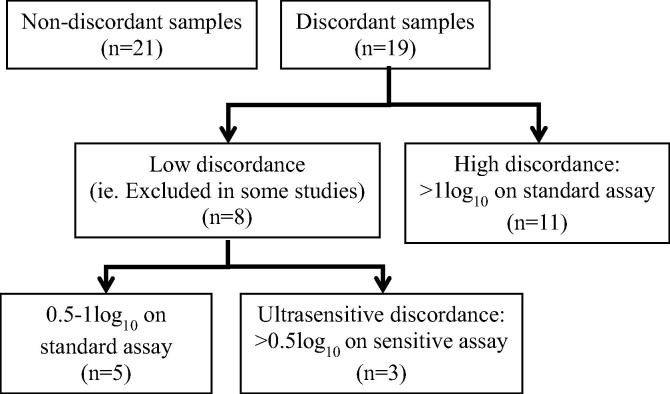
Flow-chart of groups described in analysis.

**Fig. 2:**
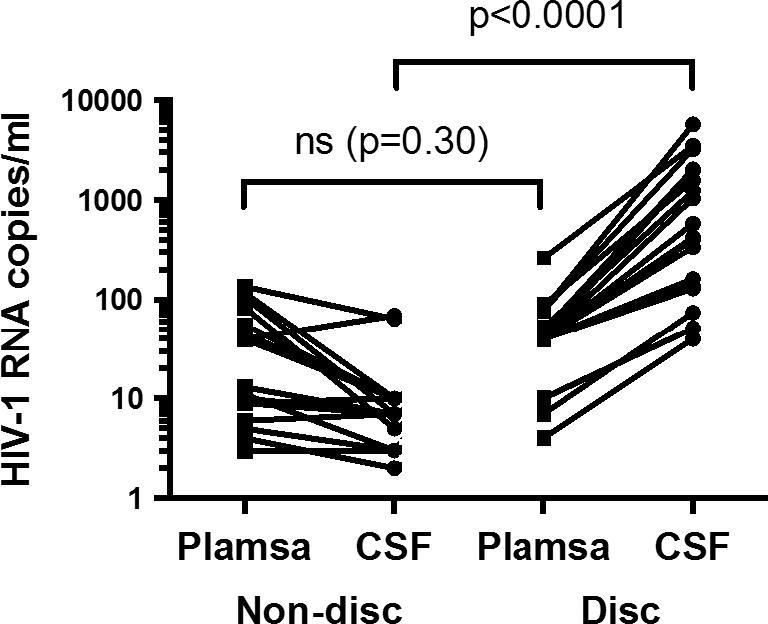
HIV-1 RNA in plasma and CSF in discordant (disc) and non-discordant (non-disc) groups.

Of 153 patients recruited in PARTITION, 40 CSF samples were analysed by cytometric bead array. Nineteen episodes of CSF/plasma discordance were identified in 18 patients (one subject had two discordant results in CSF collected at an interval of more than two years). Eleven (58%) had high discordance and eight (42%) had low discordance; of which three had ultrasensitive discordance – plasma/CSF HIV-1 RNA 4/40, <7/74 and <10/51 copies/ml (Fig. 1). Median HIV-1 RNA in plasma was <40 copies/ml (IQR <40, 52) and in CSF was median 422 copies/ml (IQR 138, 1981). Twenty-one non-discordant samples in 21 patients were selected, matched for plasma HIV-1 RNA. Nineteen (90%) had CSF HIV-1 RNA <40 copies/ml. Twelve (57%) had low CSF HIV-1 RNA demonstrated on sensitive assay; median <7 copies/ml (IQR <3, <7) Two samples had CSF detection not meeting criteria for discordance (plasma/CSF HIV-1 RNA <40/69 and 135/64 copies/ml (Fig. 2).

Demographic, clinical and laboratory parameters were similar between discordant and non-discordant samples although discordant patients tended to have a lower nadir CD4 (Table 1). Two of eight discordant patients in the LLV group had cognitive symptoms, one of which had an abnormal HIV dementia scale result. Three non-discordant patients in the LLV group had cognitive symptoms, none of which had abnormal HIV dementia scale results. Median CSF white cell count in the discordant group was 4 cells/μl (IQR <1, 17) and in the non-discordant group was <1 cell/μl (IQR <1, 1), p = 0.003. CSF white cell count was measured in 15 discordant samples, of which eight (53%) had <5 cells/μl (ie. at a level commonly considered to be normal in clinical practice) and five (33%) had <1 cell/μl. All samples in the non-discordant group had <5 cells/μl and 14 (74%) had <1 cell/μl.

### 3.2 Univariate analysis of CSF mediators

**Fig. 3:**
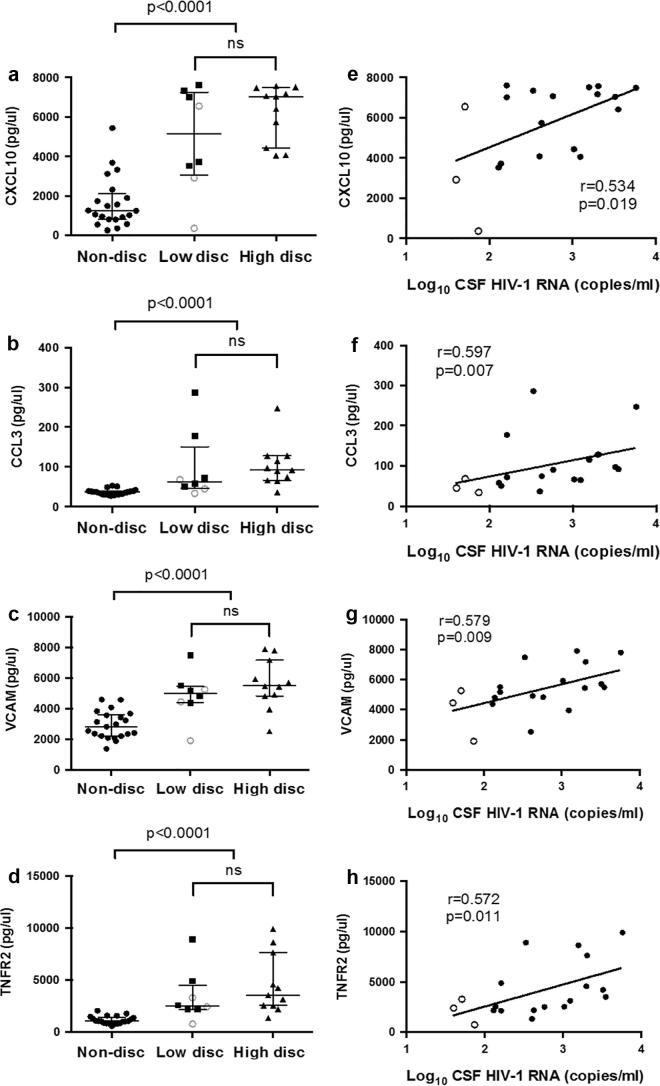
Relationship of CXCL10, CCL3, VCAM and TNFR2 concentration between groups. CXCL10, CCL3, VCAM and TNFR2 concentration (Fig. 1a–d respectively) were significantly higher in discordant versus non-discordant samples. No significant differences were observed between samples with high (CSF >1log_10_ plasma) versus low (CSF 0.5–1log_10_ plasma or ultrasensitive discordance) degrees of discordance. Samples with ultrasensitive discordance are represented with an open circle. Mediator concentrations correlated with CSF HIV-1 RNA (Fig. 1e–h). Non-disc, non-discordant; Low-disc, low-discordant; High-disc, high-discordant; ns, not significant; CXCL10, inducible protein 10; VCAM, vascular cell adhesion molecule; CCL3, monocyte inflammatory protein 1a; TNFR2, tumour necrosis factor receptor 2.

In over 80% of samples the following mediators had measurements at or below the lower limit of quantification and hence were excluded from further analysis: GCSF, GM-CSF, IFNα2, IFNβ, IL17a, IL17f, IL2, IL4, TNFα, VEGFα, MMP1, 3, 8, 9, 12 and 13. Nineteen (90%) of the remaining 21 CSF mediators were significantly higher in discordant than non-discordant samples (Table 2). This included interleukins IL-1α/β, IL-6, IL-8 and IL-10, the chemotactic CC chemokines CCL3, 4 and 5, the TNFα related proteins TNFR1, TNFR2 and TRAIL and adhesion molecules VCAM and ICAM. There were no significant differences between CSF mediator concentrations in samples with high versus low discordance (Table 2). There was a significant correlation between mediator concentrations and CSF HIV-1 RNA for nine mediators (Table 2); this was strongest for CXCL10, CCL3, VCAM and TNFR2 (Fig. 3).

### 3.3 Multivariate cluster analysis

**Fig. 4:**
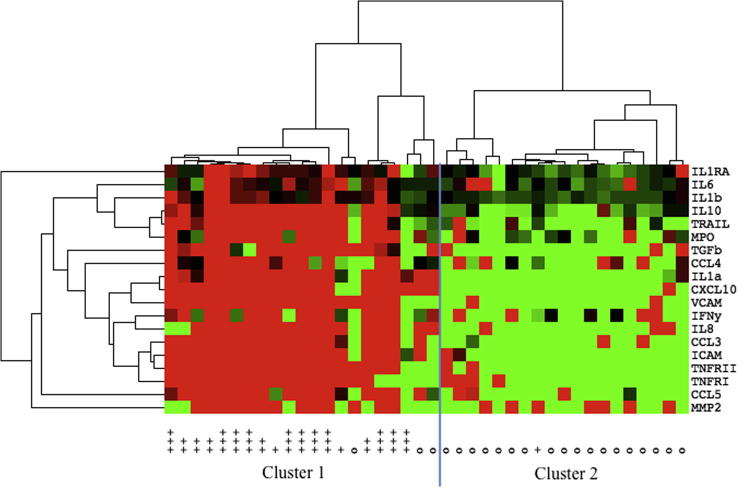
A heat map displaying the relative concentration of 19 mediators in samples with and without CSF/plasma discordance. Samples and mediators were organised via an unsupervised hierarchical clustering algorithm along the horizontal and vertical axes respectively. Twenty-one samples without discordance are labeled on the x-axis with the symbol “o”. Nineteen samples with discordance are indicated by the symbol “+”; discordant samples with CSF HIV-1 RNA >1log_10_ plasma are labeled “+++” (n = 11), CSF 0.5–1log_10_ plasma are labeled “++” (n = 5) and those with ultrasensitive discordance are labeled “+” (n = 3). Tile colour indicates relative mediator concentration: red, increased; green, decreased; black, at median concentration (for the samples). On the horizontal axis the samples segregated into two main clusters, cluster one and two, corresponding significantly to discordant and non-discordant samples respectively (p < 0.0001). Grouped in cluster one were all samples with CSF HIV-1 RNA >1log_10_ plasma, all with CSF HIV-1 RNA 0.5–1log_10_ plasma, and two of the three with ultrasensitive discordance. One subject with ultrasensitive discordance (CSF HIV-1 RNA 74, plasma <7 copies/ml) was grouped in cluster two. (For interpretation of the references to colour in this figure legend, the reader is referred to the web version of this article.)

The samples grouped into two main clusters based on relative mediator concentrations; cluster one with higher concentrations and cluster two with lower concentrations (Fig. 4). Cluster one contained 21 samples, of which 18 (86%) were discordant. Cluster two contained 19 samples of which 18 (95%) were non-discordant (p < 0.0001). Grouped in cluster one were all samples with CSF HIV-1 RNA >1log_10_ plasma, all with CSF HIV-1 RNA 0.5–1log_10_ plasma, and two of the three with ultrasensitive discordance. The single discordant sample in cluster two had ultrasensitive discordance (Plasma HIV-1 RNA <7 copies/ml, CSF 74). Two of the three samples with CSF viral escape not meeting criteria for CSF/plasma discordance were in cluster two.

The 19 CSF mediators that were found to be significantly up-regulated in discordant samples in the univariate analysis clustered into two main sets (Fig. 4). The first set included IL1β, IL6, IL10, TRAIL and MPO; the second set included TGFβ, CCL3, CCL4, CXCL10, IL1α, IL8, VCAM, ICAM, IFNγ, TNFRI and II. Three mediators (IL1RA, CCL5 and MMP2) were outlying these sets.

## 4 Discussion

In patients receiving ART, the clinical significance of CSF/plasma HIV-1 RNA discordance is poorly understood, and the relevance of low levels of discordance has not been assessed. This study found that CSF/plasma discordance was associated with the increased expression of host inflammatory mediators in the CSF in comparison to non-discordant patients. Moreover, there was no significant difference in the concentration of these mediators between patients with high and low level discordance. In addition, the mediator profiles clustered cases with both high and low discordance together and were distinct from the non-discordant patients. Taken together these findings suggest that discordance of HIV is the CSF is associated with a potentially deleterious host inflammatory response and may therefore be of significance, and that this may have implications for patients even with low levels of discordance. Longitudinal studies assessing these mediators and CSF HIV-1 RNA in conjunction with clinical outcomes are needed to better elucidate the temporal relationship between discordance, host inflammatory response, and clinical outcomes. In addition, more work is needed to examine whether discordance could be a result of an inflammatory process, rather than the cause of it.

Whether the neuroinflammation observed in association with discordance led to adverse clinical outcomes in this study is not clear. Neurological symptoms occurred both in those with and without discordance in both groups. Just one patient in the LLV group had an abnormal cognitive screen, however this is an insensitive test for milder forms of cognitive impairment. Cognitive function in HIV positive cohorts is highly variable and impairment is multifactorial, hence to identify insidious neurological damage related to long-term low-grade neuroinflammation would require larger cohorts and more detailed cognitive testing than was performed in our study [22]. In other studies raised CSF concentrations of many of these mediators have been shown to correlate with HIV-associated neurocognitive disorders, although most studies were in the pre/early-combination ART era. This subject has been reviewed extensively by Brew and Letendre [7]. So far no single mediator has been shown to have a sufficiently strong correlation with cognitive status to be a clinically useful biomarker and a combination of biomarkers may be more useful [10].

Surprisingly we did not find an association of CCL2 (MCP-1) with discordance. This chemokine is associated with chemotaxis of monocytes across the blood-brain barrier and has been associated with cognitive impairment in HIV positive populations in the pre and post-combination ART era [23]. The reasons why we found no association are unclear, but in one study higher concentrations of CCL2 were associated with both better and worse cognitive outcomes dependent on the relative concentration of other mediators, including TNFRII, and additional parameters, including nadir CD4 [10], highlighting the complex interaction of the host inflammatory response and other viral and host factors. We also expected to see an association of discordance with TNFα as CSF levels have been associated with brain injury and TNFα mRNA levels are elevated in the brain tissue of cognitively impaired HIV infected patients [24]. >80% of TNFα measurements were below the limit of quantification in our analysis, however levels of all three closely related proteins TNFR1, 2 and TRAIL were associated with discordance. CSF pleocytosis was more frequent in the discordant group, consistent with the hypothesis that discordance is associated with CNS inflammation. Of note half did not have a CSF pleocytosis and a third had no measurable white cells in CSF, suggesting that CSF white cell count alone may not be a sensitive biomarker of neuroinflammation.

Samples with higher degrees of CSF/plasma discordance tended to have higher CSF mediator concentrations. This effect was not strong and the difference between low and high discordant groups was not statistically significant for any single mediator. This is consistent with data from the CNS HIV Antiretroviral Therapy Effects Research (CHARTER) study, the largest cohort in this area, which found that although the absolute level of CSF HIV-1 RNA was not related to cognitive performance, the presence of discordant virus was [3].

When taken together the majority of samples with low CSF discordance had raised CSF mediator concentrations clustered in a similar pattern to those with high discordance. Only one low discordant subject had low CSF mediator concentrations and was grouped with the non-discordant cluster. Our data suggests that the criteria for CSF/plasma discordance used in some studies (ie. those excluding patients with <1log_10_ difference and/or <200 copies/ml in CSF) may risk type two error, and the use of these criteria in clinical practice risks missing patients with a potentially significant host inflammatory response [1], [5]. Our data suggest that the lower cut-off of 0.5log_10_ should be considered. This is consistent with our previous work demonstrating drug-resistant HIV-1 in CSF in patients with 0.5–1log_10_ discordance and <200 copies/ml in CSF [15].

Reports have suggested that CSF HIV-1 RNA detection at levels below 50 copies/ml may be associated with cognitive impairment [3], [12]. In this study, two of the three samples with ultrasensitive discordance had raised CSF mediator profiles grouped with other discordant samples. Although the numbers are small, our data support the MIND exchange consensus report recommendations that sensitive HIV-1 RNA testing should be considered in those with neurological or cognitive symptoms and less than 50 copies/ml in CSF and plasma [14]. Samples with CSF viral escape not meeting criteria for CSF/plasma discordance did not consistently have raised CSF mediator profiles. The definition of CSF viral escape could theoretically include a subject with 41 copies/ml in CSF and 39 copies/ml in plasma; such differences are likely to represent random biological and statistical variation or limitations of the assay, rather than CNS compartmentalised virus [25]. Of note, CSF viral escape has not been associated with cognitive impairment in large cross sectional studies [4], [26]. Although confirmation in larger cohorts is needed, our data suggest that patients with CSF viral escape at levels not meeting criteria for CSF/plasma discordance should be considered for sensitive testing in plasma to determine the true extent of discordance between the two compartments.

Some studies have demonstrated no association between CSF HIV-1 RNA levels and cognitive performance [27]. This may be due to the multifactorial nature of cognitive impairment in HIV rather than indicating that CSF HIV-1 detection does not have potential consequences for the brain [22]. Our data demonstrate that CSF/plasma discordance is associated with host inflammatory mediators in the CSF and support performing CSF HIV-1 RNA quantification in clinical practice. Of note the majority of discordant samples in this study had plasma HIV-1 RNA levels that were unquantifiable on standard testing and would have been classified as successfully treated on plasma testing alone. No subject had plasma HIV-1 RNA above 400 copies/ml, the level associated with long-term virological failure [25]. CSF discordance, even at low levels, requires further investigation; the indications for performing lumbar puncture solely to measure CSF HIV-1 RNA, and strategies for managing those patients found to be discordant, remain an important topic for future research.

## 5 Conclusion

CSF/plasma HIV-1 RNA discordance is associated with a potentially damaging neuroinflammatory process. Patients with discordance at lower levels (ie. 0.5–1log_10_) should also be investigated as mediator profiles were similar to those with discordance >1log_10_. Sensitive testing may have a role to determine whether ultrasensitive discordance is present in those with low level CSF escape.

## Funding

SN received a research award for this work from the British HIV Association. SN is a MRC Clinical Training Fellow supported by the North West England Medical Research Council Fellowship Scheme in Clinical Pharmacology and Therapeutics, which is funded by the Medical Research Council (Grant No. G1000417/94909), ICON, GlaxoSmithKline, AstraZeneca and the Medicines Evaluation Unit. BDM is an NIHR Academic Clinical Lecturer and received funding as part of a NIHR Doctoral Research Fellowship.

## Conflict of Interest

None.

## Figures and Tables

**Table 1:** Subject characteristics in 19 episodes of CSF/plasma discordance >0.5log_10_ versus 21 without discordance.

	Discordant (n = 19)	Non-discordant (n=21)	p value
Age, median years (IQR)	47 (44, 57)	47 (41, 53)	0.577
Gender male, n (%)	15 (78.9)	15 (71.4)	1.00
Ethnicity, n (%)			0.408
White MSM	8 (42.1)	12 (57.1)	
Black heterosexual	6 (31.6)	3 (14.3)	
Other	5 (26.3)	6 (28.6)	

CD4			
Current, median cells/μl (IQR)	380 (319, 490)	464 (359, 741)	0.189
Nadir, median cells/μl (IQR)	31 (27, 79)	112 (34, 283)	0.102

Time since HIV diagnosis, median years (IQR)	13.0 (9.3, 18.0)	8.0 (4.5, 16.5)	0.087

ART at sampling, n (%)			0.306
PI/r	17 (89.5)	13 (61.9)	
NNRTI	2 (10.5)	7 (33.3)	
NRTI	17 (89.5)	18 (85.7)	
RAL	5 (26.3)	2 (9.5)	
MVC	2 (10.5)	3 (14.3)	

CPE score (2010), median (IQR)	7 (7, 8)	7 (6, 9)	0.903
Self reported adherence <95%, n (%)	2 (10.5)	2 (9.5)	1.00
Indication for LP, n (%)			0.139
Research LP (LLV group)	8 (42.1)	8 (38.1)	
Cognitive symptoms	5 (26.3)	2 (9.5)	
Headache	0 (0.0)	3 (14.3)	
Suspected CNS infection	1 (5.3)	2 (9.5)	
Follow up previous CNS infection^a^	3 (15.8)	0 (0.0)	
Abnormal neurological exam	1 (5.3)	4 (19.0)	
Other	1 (5.3)	2 (9.5)	

a CNS toxoplasmosis, EBV encephalitis and VZV encephalitis. IQR, interquartile range; MSM, men who have sex with men; IVDU, intravenous drug user; LP, lumbar puncture; CNS, central nervous system; CSF, cerebrospinal fluid; CPE, CNS penetration effectiveness score (2010); ART, antiretroviral therapy; PI/r, ritonavir boosted protease inhibitor; NNRTI, non nucleoside reverse transcriptase inhibitor; RAL, raltegravir; MVC, maraviroc; RNA, ribonucleic acid.

**Table 2:** Relationship of mediator concentrations between groups and correlation with CSF HIV-1 RNA.

	All subjects			Discordant subjects			Correlation with CSF HIV-1 RNA	
	Discordant (n = 19)	Non-discordant (n = 21)	p value	Low discordant (n = 8)	High discordant (n = 11)	p value	Spearman r	p value
CCL2	1271 (872.8, 1582)	944.5 (792.9, 1322)	0.305	1139 (691.6, 1562)	1271 (878.8, 1678.0)	0.753	0.284	0.240
CCL3	74.5 (58.0, 128.0)	36.8 (30.8, 39.6)	**<0.001**	63.0 (46.6, 150.8)	92.0 (66.5, 128.0)	0.265	**0.597**	**0.007**
CCL4	23.5 (16.0, 59.0)	14.5 (13.0, 18.8)	**0.004**	17.8 (15.3, 48.9)	26.5 (22.5, 66.5)	0.201	**0.506**	**0.027**
CCL5	57.5 (25.0, 190.5)	14.5 (11.5, 22.3)	**<0.001**	33.8 (22.1, 157.3)	85.8 (31.5, 281.5)	0.282	**0.523**	**0.022**
CXCL10	6553 (4058, 7342)	1249 (812.8, 2114)	**<0.001**	5136 (3067, 7260)	7038 (4439, 7480)	0.232	**0.534**	**0.019**
ICAM	296.0 (176.8, 622.5)	71.5 (62.3, 93.8)	**<0.001**	286.0 (161.2, 525.7)	325.0 (204.0, 774.0)	0.528	0.449	0.054
IFNy	40.0 (32.0, 48.3)	29.5 (24.5, 34.9)	**0.001**	39.9 (32.1, 46.1)	41.5 (32.0, 53.3)	0.762	0.233	0.338
IL10	26.5 (22.5, 33.0)	17.5 (16.5, 19.5)	**<0.001**	25.8 (21.3, 28.1)	31.0 (22.5, 37.8)	0.455	**0.471**	**0.042**
IL1a	53.0 (39.8, 62.0)	20.5 (18.0, 30.3)	**<0.001**	47.1 (36.6, 60.9)	53.0 (43.5, 63.0)	0.362	**0.478**	**0.038**
IL1b	9.0 (8.0, 11.0)	7.0 (7.0, 7.8)	**<0.001**	8.8 (8.0, 9.9)	9.5 (8.0, 14.5)	0.453	0.269	0.265
IL1RA	15.0 (13.8, 17.5)	13.5 (12.5, 14.0)	**0.001**	15.0 (13.5, 15.5)	16.0 (14.3, 24.8)	0.264	0.348	0.144
IL6	9.0 (8.0, 10.5)	7.3 (6.6, 8.3)	**0.005**	8.5 (7.9, 10.8)	9.0 (7.9, 10.5)	0.953	0.219	0.367
IL8	125.5 (96.0, 169.5)	63.0 (53.0, 79.4)	**<0.001**	119.4 (79.9, 145.4)	128.5 (104.0, 117.3)	0.636	0.322	0.179
MMP2	1234 (959.6, 1335)	941.4 (757.7, 1154)	**0.014**	1186 (910.6, 1308)	1263 (959.6, 1485)	0.429	0.437	0.061
MMP7	35.1 (26.7, 46.0)	34.1 (22.1, 39.5)	0.634	34.8 (28.8, 43.7)	35.5 (25.7, 46.9)	0.954	0.265	0.272
MPO	23.0 (17.5, 67.8)	16.0 (13.0, 17.6)	**0.001**	21.6 (16.4, 27.8)	29.5 (21.3, 117.0)	0.134	0.414	0.078
TGFb	82.8 (65.5, 99.8)	55.0 (47.0, 65.1)	**0.003**	91.9 (68.6, 134.1)	82.8 (64.0, 94.0)	0.236	−0.097	0.692
TNFR1	11,788 (8992, 14,874)	7761 (7461, 9346)	**0.001**	11,267 (9256, 13,491)	11,788 (8444, 18,161)	0.636	0.424	0.071
TNFR2	3131 (2195, 4891)	1032 (815.8, 1402)	**<0.001**	2477 (2159, 4492)	3514 (2526, 7629)	0.302	**0.572**	**0.011**
TRAIL	42.5 (36.5, 51.5)	26.9 (29.4, 31.8)	**<0.001**	40.3 (35.4, 46.3)	45.3 (37.3, 52.0)	0.341	**0.480**	**0.038**
VCAM	5263 (4458, 5935)	2222 (2831, 3629)	**<0.001**	4995 (4400, 5452)	5508 (4835, 7190)	0.232	**0.579**	**0.009**

Nineteen of 21 CSF mediators were significantly (p < 0.05) higher in discordant than non-discordant subject in univariate analysis. There were no significant differences between CSF mediator concentrations in samples with high (CSF HIV-1 RNA >1log_10_ plasma) versus low (0.5–1log_10_ plasma or ultrasensitive discordance) discordance (Table 2). Nine CSF mediator concentrations correlated with CSF HIV-1 RNA.

CSF, cerebrospinal fluid; RNA, ribonucleic acid. Values are median pg/μl (IQR). P values are by Mann-Whitney-U or Spearman r correlation.
